# Prevalence and parasite burden of oocysts in captive and free-living saffron finches, *Sicalis flaveola*

**DOI:** 10.1590/S1984-29612024029

**Published:** 2024-06-17

**Authors:** Francisco Carlos Rodrigues de Oliveira, Samira Salim Mello Gallo, Taynara Kerolayne Santos Elizeu, Nicole Brand Ederli

**Affiliations:** 1 Laboratório de Sanidade Animal, Universidade Estadual de Norte Fluminense Darcy Ribeiro – UENF, Campos dos Goytacazes, RJ, Brasil; 2 Instituto do Noroeste Fluminense de Educação Superior, Universidade Federal Fluminense – UFF, Santo Antônio de Pádua, RJ, Brasil

**Keywords:** OoPg, Passeriformes, oocysts, feces, OoPg, Passeriformes, oocistos, fezes

## Abstract

The saffron finch, *Sicalis flaveola*, a passerine bird, can be found in nearly all Brazilian territory and is also raised in captivity. The objective of this work was to determine the prevalence and load of oocysts in captive saffron finches in the municipality of Campos dos Goytacazes, state of Rio de Janeiro and in free-living saffron finches in the municipality of Eugenopolis, state of Minas Gerais. In this analysis, 30 captive and 30 wild birds were assessed. Feces eliminated in a 24-hour period were collected and weighed to determine the number of oocysts per gram of feces (OoPG). Statistical analyses were performed using Microsoft Excel and GraphPad Prism Software. All birds in the present study were positive for one or more species of coccidia. Captive birds had a mean total oocyst count higher than that of wild birds. No significant differences in OoPG counts were observed when comparing males and females or captive and wild birds. We can conclude that due to the fact that birds both eat and defecate in their cages, it is essential to keep them as clean as possible, since captive birds have a higher prevalence of coccidia.

## Resumo

Canário-da-terra, *Sicalis flaveola*, uma ave passeriforme, está presente na natureza em praticamente todo o território brasileiro, além de ser criada em cativeiro. O objetivo deste trabalho foi determinar a prevalência e carga de oocistos em canários-da-terra de cativeiro, no município de Campos dos Goytacazes, estado do Rio de Janeiro. E de canários-da-terra de vida livre no município Eugenopolis, estado de Minas Gerais. Para isso, foram utilizadas 30 aves de cativeiro e 30 de vida livre. Fezes eliminadas durante 24h foram coletadas e pesadas para a realização da contagem de oocistos por grama de fezes (OoPG). Análises estatísticas foram feitas com o auxílio do Software Microsoft Excel e Graphpad Prism. Todas as aves do presente estudo estavam positivas para uma ou mais espécie de coccídio. As aves de cativeiro apresentaram média de contagem total de oocistos maior do que as aves de vida livre. Não foi observada diferença significativa nas contagens de OoPG com relação ao sexo e à origem das aves de cativeiro ou de vida livre. Pode-se concluir que, devido ao fato das aves comerem e defecarem em suas gaiolas, é essencial mantê-las as mais limpas possíveis, uma vez que as aves em cativeiro apresentam maior prevalência de coccídios.

The saffron finch, *Sicalis flaveola*, is a small, bright yellow neotropical passerine native to South America, ranging from Argentina to Venezuela; introduced populations also exist in other locales, including Puerto Rico, Jamaica, and Hawaii. The birds travel in pairs or small flocks, forage for insects and seeds on the ground, and visit feeders in suburban backyards (Rising, 2011).

Coccidia are a highly diverse subclass of protozoan obligate intracellular parasites of domestic and wild animals (Ruggiero et al., 2015; Fayer, 1980). Although little is known about avian coccidia in wild birds worldwide, in captive birds, low levels of coccidia are common and harmless in most bird species (Greiner & Ritchie, 1994), but severe infections are known to cause severe endemic disease in captive birds (Schoener et al., 2013).

Until 2011, only *Isospora sicalisi* and *Isospora cetasiensis* were reported in *S. flaveola* (Coelho et al., 2011); however, more recently, *Isospora bertoi* and *Eimeria flaveola* were reported in birds of this species that lived free between the border of the Northwest of the State of Rio de Janeiro and Zona da Mata of Minas Gerais, Brazil (Gallo et al., 2022; Oliveira et al., 2023). Recently, *Cryptosporidium andersoni* was diagnosed in a saffron finch from a commercial establishment in northern Rio de Janeiro (Oliveira et al., 2022).

This study was conducted to determine the prevalence and load of oocysts in the feces of wild saffron finch *S. flaveola* from the state of Minas Gerais and captive saffron finch in Campos dos Goytacazes, in the state of Rio de Janeiro.

A total of 60 adults saffron finches, *S. flaveola*, were included in the study, of which 30 were raised in breeding cages and commercial establishments in the municipality of Campos dos Goytacazes, Rio de Janeiro, Brazil. The other 30 birds were free-living and inhabited peri-urban and rural regions of the municipality of Eugenopolis, Minas Gerais and were captured with mist nets. After capture, the birds were individually housed for 24 hours in cages with water and food *ad libitum*. This study was approved by the Biodiversity Authorization and Information System (SISBIO) under protocol n° 78,016–1/2022, and all experimental protocols were approved by the ethics committee for the use of animals (protocol n° 523). The distinction between the sex of adult birds was possible because they present marked sexual dimorphism in the coloration of their plumage (Benítez Saldívar & Massoni, 2018; Cruz-Bernate et al., 2023).

Feces from 24 hours after capture were collected from the bottom of each cage and were placed in 15 mL tubes, identified, placed in an isothermal box with ice and immediately transported to the Núcleo de Pesquisas Avançadas em Parasitologia (NUPAP) at the Universidade Estadual do Norte Fluminense Darcy Ribeiro (UENF) in the Municipality of Campos dos Goytacazes, Rio de Janeiro, Brazil.

A portion of the fecal samples from all captured birds were filtered through double gauze, mixed with 2.5% potassium dichromate (K_2_Cr_2_O_7_), placed in a Petri dish and incubated at 23–28 °C until 70% of the oocysts were sporulated. Oocysts were retrieved by the flotation method with Sheather’s sugar solution and examined microscopically using the method described by Duszynski & Wilber (1997).

Fecal oocyst counts were performed by Wisconsin sugar flotation method with oocysts recovery at 20 min. The number of oocysts per gram of feces (OoPG) was calculated as the total count of oocysts divided by the total weight of the sample in grams (Tookhy et al., 2022). The mean number, standard deviation, minimum and maximum value of observed oocysts were calculated using Microsoft Excel 2013™ software (Microsoft, Redmond, WA, USA). Student's t test was the statistical method used to compare means between groups of captive and free-living birds. For this, the GraphPad Prism 5™ program (GraphPad Software, SD, USA) was used and 95% confidence intervals were computed.

**Figure 1:**
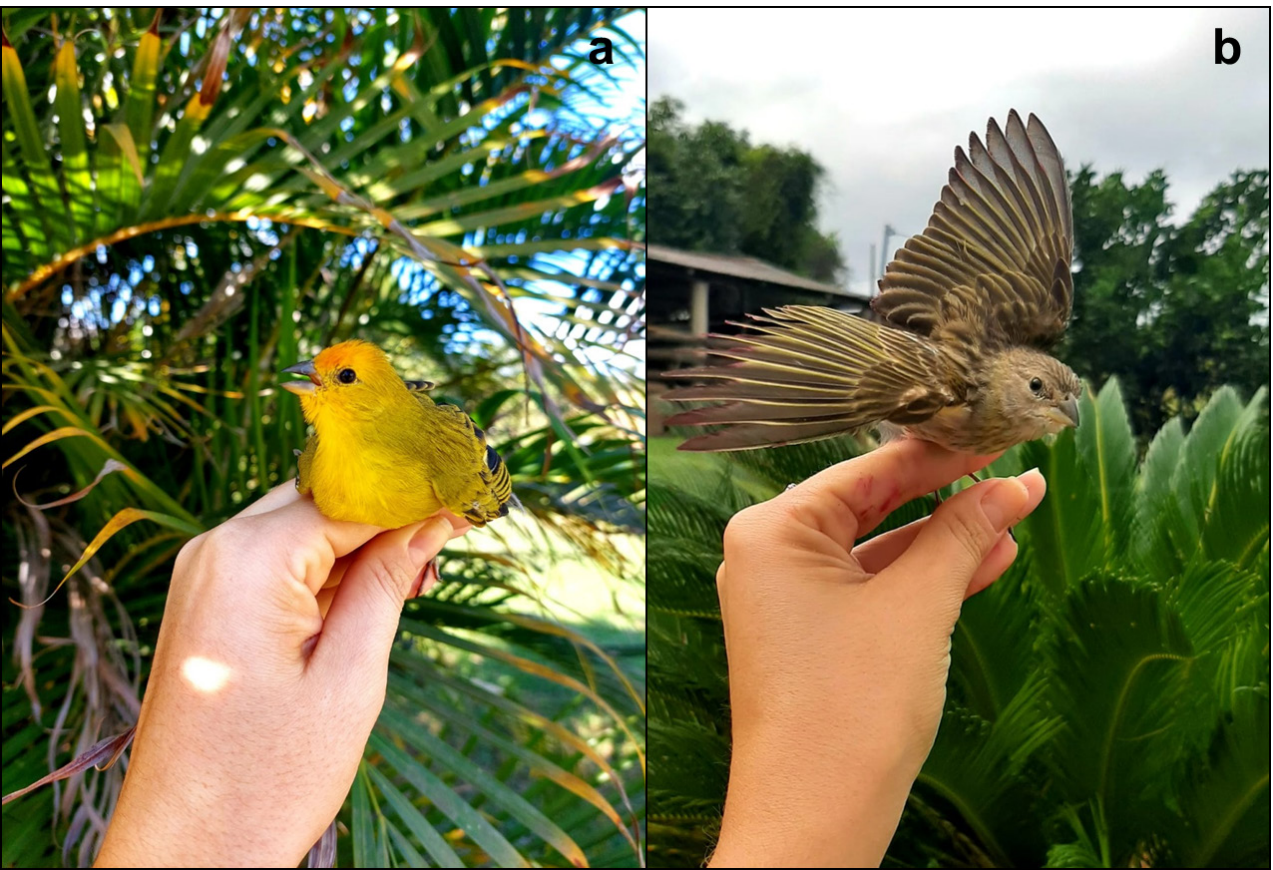
Wild saffron finches, *Sicalis flaveola*, captured in Eugenopolis, state of Minas Gerais, Brazil. In **a**, male specimen and in **b**, female specimen.

Among the captive birds, 26 (87%) had sexual dimorphism, where 21 (70%) were males and five (17%) were females; however, in four (13%) of these birds, it was not possible to determine the sex (Table 1). Of the wild birds (Figure 1), nine (30%) were identified as males, 14 (47%) as females, and in seven (23%), it was not possible to identify the sex of the captured bird (Table 2).

**Table 1:** Oocysts per gram of feces (OoPG) of captive saffron finches, *Sicalis flaveola*, in relation to sexual dimorphism.

Birds	Sex	Grams of feces	Total count	OoPG
C1	Male	3.24	1,334	412
C2	Male	3.40	39	11
C3	Female	3.20	186	58
C4	Female	1.96	**4**	**2**
C5	Female	3.65	530	145
C6	Male	1.92	2,881	1,504
C7	Male	2.51	1,296	516
C8	Male	1.53	146	95
C9	Male	1.54	2,866	1,867
C10	-	3.23	5,535	1,716
C11	-	4.04	6,616	1,638
C12	-	4.41	2,210	502
C13	Male	1.71	264	155
C14	Male	1.68	200	119
C15	Male	1.78	930	522
C16	Male	1.50	1,556	1,037
C17	Male	1.41	1,755	1,245
C18	Male	2.20	3,026	1,379
C19	Male	1.93	251	130
C20	-	2.59	2,224	860
C21	Male	4.72	**16,886**	3,578
C22	Male	5.21	2,001	384
C23	Male	2.50	133	53
C24	Male	1.75	691	395
C25	Male	2.33	10,022	**4,311**
C26	Male	**6.28**	15,005	2,391
C27	Male	1.64	46	28
C28	Female	2.45	34	14
C29	Male	**1.10**	781	710
C30	Female	4.79	348	73
**MEAN**		**2.74**	**2,660**	**862**

**Table 2:** Oocysts per gram of feces (OoPG) of wild saffron finches *Sicalis flaveola* in relation to sexual dimorphism.

Birds	Sex	Grams of feces	Total count	OoPG
W1	Female	2.25	6	**3**
W2	Female	1.63	739	455
W3	-	2.62	4,817	1,842
W4	-	1.50	1,086	724
W5	-	2.48	1,026	415
W6	-	2.44	69	28
W7	-	1.73	677	392
W8	Female	3.00	**10,049**	3,350
W9	Female	3.70	1,126	304
W10	Male	3.45	1,420	412
W11	Male	**6.00**	619	103
W12	Female	3.80	67	18
W13	-	2.44	8,223	**3,377**
W14	Female	1.75	2,336	1,335
W15	Female	1.85	**5**	3
W16	-	2.00	5,435	2,718
W17	Female	1.50	3,281	2,187
W18	Female	1.95	6,298	3,230
W19	Male	1.99	5,921	2,983
W20	Female	1.90	3,085	1,624
W21	Female	0.78	690	885
W22	Male	0.76	377	499
W23	Male	0.61	794	1,312
W24	Female	1.95	116	59
W25	Male	1.41	62	44
W26	Female	**0.50**	49	98
W27	Male	1.51	38	25
W28	Male	1.23	63	51
W29	Female	1.53	1,523	999
W30	Male	1.18	78	66
**MEAN**		**2.05**	**2,003**	**985**

All birds in the present study were positive for one or more species of coccidia, thus indicating a prevalence of 100%. From captive birds, it was possible to collect an average of 2.74 (1.10-6.28) grams of feces, with a total oocyst count ranging from four to 16,886 and an average of 2,660 oocysts shed in 24 hours. The OoPG count ranged from 2 to 4311 with a mean of 862 (Table 1).

From wild birds kept in cages for 24 hours, it was possible to collect an average of 2.05 (0.5-6.0) grams of feces. In these feces, 5 to 10,049 oocysts were counted per bird, with an average of 2003 shed oocysts. For this group of birds, the OoPG ranged from 3 to 3377 with a measured average of 985 oocysts (Table 2).

No significant differences were observed in the OoPG count of the saffron finch based on the sex of birds raised in captivity (P=0.0967) or captured from the wild (P=0.3731), or when the prevalence of all birds was analyzed as a single group (P=0.7683). (Table 3).

**Table 3:** Oocysts per gram of feces (OoPG) of male and female saffron finches, *Sicalis flaveola*.

Birds	n^1^	OOPG				P value^2^
		Mean	Standard Deviation	Minimum	Maximum	
Captivity						
Male	21	992	1,189	11	4,311	0.0967
Female	5	58	57	2	145	
**Wild**						
Male	9	610	981	25	2,983	0.3731
Female	14	1,039	1,171	3	3,350	
**Captivity and Wild**						
Male	30	878	1,128	11	4,311	0.7683
Female	19	781	1,090	2	3,350	

1 Number of animals;

2 Student's t test with 95% confidence interval.

No significant difference was observed in the OoPG counts (P=0.6689) of the saffron finch by origin (captive *vs.* wild; Table 4).

**Table 4:** Frequency of oocysts per gram of feces (OoPG) in relation to the origin of saffron finches, *Sicalis flaveola*.

Birds	n^1^	OoPG				P value^2^
		Mean	Standard deviation	Minimum	Maximum	
Captivity	30	862	1,054	2	4,311	0.6689
Wild	30	985	1,125	3	3,377	

1 Number of animals;

2 Student's t test with 95% confidence interval.

In captive and wild, in general, prevalences of 9% for *Isospora sicalisi*, 47% for *Isospora cetasiensis* and 7% for *Isospora bertoi* were identified. A prevalence of 37% of oocysts of the species *Eimeria flaveola* was also observed.

*Isospora sicalisi* oocysts had a prevalence of 32% in captive birds and 1% in wild birds, while *I. cetasiensis* oocysts had a prevalence of 68% in captive birds and 39% in wild birds. In wild birds, 2% of *I. sicalisi* oocysts and 2% of *I. cetasiensis* oocysts with polar granules were observed, a morphological characteristic not yet reported for these species. For oocysts of *I. bertoi* and *E. flaveola*, prevalences of 9% and 48%, respectively, were calculated in wild birds.

The high prevalence rate (100%) of oocysts in both captive and wild birds in the present study did not corroborate the findings of Coelho et al. (2011), who reported a prevalence of 69% in captive *S. flaveola*. This may support the hypothesis of Alasadiy et al. (2022): a greater prevalence of these coccidia is related to climatic conditions such as humidity and temperature, which under ideal conditions are more suitable for the development of parasitism than the sex of the host. These same researchers reported that captive birds had a greater chance of infection because they are confined to small areas with larger groups of individuals, contrary to what Rising (2011) observed in nature, and this increases the possibility of ingestion of large numbers of oocysts. This finding was supported by the results of our study, although no significant difference by group was detected in this analysis.

*Isospora bertoi* and *E. flaveola*, recently described in wild birds (Gallo et al., 2022; Oliveira et al., 2023), were not observed in the feces of birds from Campos dos Goytacazes. Although *Cryptosporidium* spp. was also not observed, we cannot infer that the birds in this project did not harbor this parasite in their feces since the coprological analyses were performed by common optical microscopy, which is not the appropriate diagnostic method for detecting this parasite. *Isospora sicalisi* and *I. cetasiensis*, two species described by Coelho et al. (2011) in a study of captive *S. flaveola*, 26 birds were assessed and 69% (18) were infected by *I. cetasiensis* and 12% (3) by *I. sicalisi*. Our research corroborates the study by Coelho and collaborators, since among the 30 captive birds in our study, *I. cetasiensis* was more prevalent (68%) compared to *I. sicalisi* (32%). Similarly, we found that *I. cetasiensis* was more prevalent (39%) in wild birds than *I. sicalisi* (1%).

No significant differences were observed in the OoPG count of the saffron finch when assessed by sexual dimorphism, either among captive (Table 1) or wild birds (Table 2), or overall (Table 3). According to Brown et al. (2010), the general prevalence of infection does not seem to be influenced by the sex of the host, since the infection is significantly affected by direct contact with feces, type of food, use of parasiticides, type of enclosure and how often the cages are cleaned. In wild birds, however, infection can be influenced by the loss of natural habitats, population accumulation, illegal seizures, availability of food, shelter and climatic conditions (Friend & Franson, 1999; IUCN, 2002; Rising, 2011).

In the present study, no significant differences were observed in the OoPG counts (P=0.6689) of the saffron finch when the origin of the birds was considered (captive *vs.* wild; Table 4). Research carried out by Costa et al. (2010) also demonstrated that wild and captive animals commonly had coccidia, but a significant difference in prevalence between them was not reported. Other authors, such as Quiroga et al. (2000) and McQuistion (2000), reported that captive and wild passerines can be infected by coccidia regardless of their origin. Although no significant difference was detected by origin, it is still important to understand the impact on the infections caused in wild birds since little is known about the morphology of the oocysts of these birds (Greiner, 2008); other species (Gallo et al., 2022; Oliveira et al., 2023) that have not yet been reported may be circulating among wild birds and may affect captive birds.

We can conclude that the total average count of oocysts was higher in captive birds due to the fact that they eat and defecate in their cages and are always in contact with infective oocysts. Inadequate management favors a greater prevalence of coccidia in captive birds, therefore, regular cleaning is an important part of bird's health and wellness. Furthermore, although coccidiosis may be a primary factor in mortality, its importance is also related to increasing the host's susceptibility to other diseases, thus reducing the chances of survival during periods of stress, both in the wild and in captivity.
